# The Practitioner's Relaxations and Hobbies

**Published:** 1912-03-30

**Authors:** 


### THE PRACTITIONER'S RELAXATIONS AND HOBBIES.

### Nature Notes.—IX.

Ratting—The Value of Stoats—Under the Cornstack—A Fight—Eremites—A Rat Hunt—Up in the Hedge A Popular Superstition.

Fear that plague is in England has attracted attention to rats. They exist in great numbers in the downland. There they find abundance of food and fewer foes than in most districts, because much corn is grown, population is scanty, and game is extensively preserved. Where people are, few rats' haunts escape notice; where game is preserved, keepers kill down "the vermin." This term includes rats, and the keepers wage war without end against them; but rats are prolific and wary of traps. Hawks and owls, foxes and badgers, stoats and weasels are less prolific, and at least as easy to trap as rats, so easier to keep down. All are enemies of rats in varying degrees, and the rats gain from the destruction of these foes more than they lose from the efforts of the keepers.

Since rats are as destructive to game as any vermin, I incline to believe that the good done by stoats and weasels in destroying them, except a big head of rabbits be desired, should more than repay the game preserver the toll they take of his game. There can be no doubt of the good the stoat tribe do to the corn-grower.

Yet farmers have told me that a few rats are not entirely an evil in a corn-stack, because they kill and eat the mice. Mice are wasteful feeders, spoiling more corn than they eat, and increase so greatly in a rat-free rick that they do more harm than a moderate number of rats would do. The balance of Nature is delicate.

Rats will kill and eat any living thing less strong than themselves. They are fierce fighters, and well armed. Their sharp incision teeth cut to the bone, and enable a full-grown rat to hold its own even against a stoat if, as in a hole, there be no room for the stoat to gain an advantage from superior agility.

I got to know when the thrashers would come to a corn-stack that stood below the downs, and that showed signs of the presence of many rats. I took my terriers there towards the end of the threshing. There were fewer rats than I expected. I noticed that all that came to hand were full-grown, and many of them exceedingly large. The dogs caught none half-grown; the only small rats were eight, still blind and naked, that I found in a nest. Just before the last sheaves were tossed on to the machine two stoats sprang from the stack and escaped into a hedge. We killed thirty-four rats of large size and none small, except the eight little ones I have mentioned. My explanation is: after the stoats had taken quarters in the rick they killed off the smaller rats but left the big ones alone, not caring to face fights of doubtful issue.

Outside a hole a stoat will successfully attack a full-grown rat. One day, as I was driving between crops on a slope of the downs, I saw a rat and a stoat fighting in the road in front of me. The stoat moved with admirable agility, springing to this side of the rat and that till, in a few seconds, it got hold. Both animals rose on their hind legs, like dogs fighting, but the rat was soon twisted over and dragged into the edge of the corn out of my sight. I got down, and found the rat lying at the point of death, but I did not see the stoat again; it had fled at my approach.

Foxes and badgers will eat rats. The badgers particularly seem to like them. Captive badgers I have kept used to leave the rat's skin nearly whole, licked clean and turned inside out.

Though many rats are found in places that afford a large supply of food, such as stacks and barns and neglected slaughter-yards, I do not think it would be correct to write that they "congregate" in such places. Essentially they are solitary animals, each one fending for itself. All through the year, but especially in summer, rats can be found living in the hedgerows, by the banks of rivers, in fields of roots, and in the cornfields before the corn is cut. In such places usually each has its own earth. The earths are much on the same plan; there will be three or four entrances leading to a chamber that contains a nest of hay, leaves, pieces of paper, or other soft material. I have dug out dozens of earths, and usually have found but one rat in each, sometimes two, then always a male and female no doubt together for sexual reasons, very seldom three. Litters of quite young rats may be found in these earths, but so soon as they are half-grown the young go off to fend for themselves, and become solitary. I do not think old rats have much affection for their young. I have never seen an old rat leading her litter as an old stoat leads hers. But the stoat is an exceptionally affectionate mother.

My experience of rats is great, because for many years I have hunted them regularly with terriers in the hedgerows and on the banks of the rivers and canals I have dwelt near. With a good team of terriers it is a sport not to be despised. The best sport is obtained in summer evenings. I take three or four terriers, and draw likely spots or places where I have noticed the runs or tracks or other sign of rats in riding by. Usually the dogs soon pick up a line and run it into a thicket of herbage under the hedge. There the increased excitement of the pack shows the rat is on foot, and a merry hunt ensues through grass and nettles and the undergrowth of the hedge. In its search for food the rat may have gone a couple of hundred yards or more away from its earth. It is sure to try and get back to it. Baffled and overtaken in its efforts to reach home, it may be caught in the hedge, but often after more or less hunting it gets to ground. A terrier hunts up to the earth and marks, and speedily the whole pack is digging and tearing at the entrances, frantically eager. I help them with a strong stick, and they soon open up the earth, for these burrows usually run so near the surface that it is not difficult to push a stick through the ground into the pipe. Often the dogs will wind the rat through the ground, and dig straight down on to it. When the earth is opened up the rat is either caught in its hole, pulled out, and torn to pieces, or it makes a bolt and has to be hunted again along the hedgerow. Routed from its earth, the rat seldom seems to know of another burrow, unless there be one within a very few yards, but runs the hedgerow, taking refuge in stoles or hollow places under the roots of trees. Often it will climb the hedge and sit silent and still some feet above the ground. This manœuvre leads to pretty dog-work.

An experienced terrier, having lost the rat, will get a little from the hedge, run along the leeside, hold its nose to the breeze blowing through, so catch the scent, and work up wind and find the rat above him. I have never found a store of food in a rat's burrow. I do not think they take thought for the morrow. In burrows in or near cornfields I sometimes find ears of corn with grains still in them, but these have been carried in for present consumption or to make a nest, not as a store.

The low type of the rat's brain would not lead one to expect of them the intelligence with which they are credited. Nor have I seen evidence of such intelligence. I do not believe tales of rats combining to steal eggs or to carry off articles too heavy for the strength of an individual. They are too quarrelsome to combine, even had they the sagacity. They, however, learn to avoid traps and possibly to suspect poison after bait has been laid a few times near their haunts.

I have never found reliable evidence of a migration of rats in a mass. If food supply is removed rats necessarily move also, but not in a band. They drift off individually down the hedgerows and scatter over the neighbourhood; many of the younger falling victims to the famine of the older. When hunted they do not show cunning and resource as a fox. The most cunning trick I have seen is, when hunted on the banks of a stream, to take the water, sink themselves till the tail points to the bottom of the river, and only nostrils and whiskers are above the surface, and, keeping this position by very slight movements of the paws, thus to drift with the stream till they judge it safe to come to the surface and swim ashore. The land-rat is as able in the water as his pretty cousin, the water-rat. A man may safely put his hand down a rat's hole and pull out the rat, though many will not believe it. I have done so scores of times, and have not been bitten half-a-dozen times. The hole must be dark though. If the rat can see the hand approaching him it will rush at it and bite savagely.

